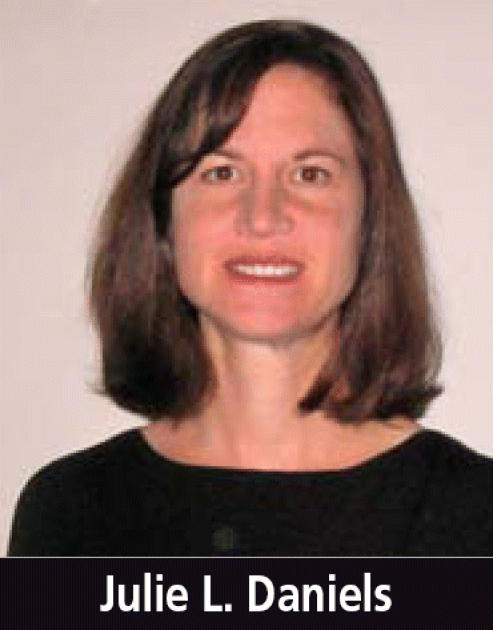# Guest Editorial: Autism and the Environment

**DOI:** 10.1289/ehp.114-a396

**Published:** 2006-07

**Authors:** Julie L. Daniels

**Affiliations:** University of North Carolina School of Public Health, Chapel Hill, North Carolina, E-mail: juliedaniels@unc.edu

Speculation that the environment plays a role in the development of autism primarily comes from two observations: *a*) although concordance among monozygotic twins is high, it is not perfect, and a specific “autism gene” or set of genes has not yet been identified; and *b*) the prevalence of autism is higher than previously thought—if it is rising, the rise might be associated with a shift in the environment.

Autism is a complex neurodevelopmental disorder defined by impaired social interaction, communication deficits, restricted interests, and repetitive behavioral patterns. These traits can range from mild to very severe, and may be accompanied by cognitive impairment and other comorbidities. The autism spectrum disorder (ASD) classification includes three disorders: autistic disorder, Asperger disorder, and pervasive developmental disorder not-otherwise-specified; however, there is no evidence that these diagnostic labels represent etiologically homogeneous groups.

The high concordance rates among monozygotic twins and recurrence in families support a strong genetic contribution to ASDs (Bailey et al. 1995; Folstein et al. 1977; Ritvo et al. 1985; Steffenburg et al. 1989). There is also a growing acceptance that subtle autism-like traits, such as atypical communication and aloof personality style, more commonly cluster in the nonautistic family members of individuals with autism than in the general population (Murphy 2000). The segregation of the milder traits in family members may indicate the presence of some, but not all, of the factors (genetic or environmental) necessary to develop an ASD.

To date, no specific genes or combination of genes have been consistently associated with autism. Discrepancies in gene-discovery studies might be, in part, because ASDs result from a variety of gene–gene and gene–environment combinations. Despite the lack of a specific genetic mechanism, most researchers agree that the etiology of autism is heterogeneous and polygenetic, and for some susceptible individuals, might involve environmental triggers.

Much of the concern surrounding environmental factors and autism comes from the perception that the prevalence of autism is increasing. There has clearly been a rise in the number of individuals who are actually diagnosed with an ASD; however, there are few systematically collected data in the same population over time that can be used to evaluate true prevalence rate trends (Fombonne 2003; Rutter 2005). Many factors could contribute to increases in prevalence estimates over time, including changes in diagnostic criteria, increasing availability of specialized diagnostic tools, improved case ascertainment, and true changes in the prevalence.

Real shifts in prevalence could result from environmental changes. Systematically monitoring temporal ASD prevalence trends in the same population over time is a necessary step to identifying true changes in prevalence. However, ecologic associations between environmental changes and rising autism rates are not sufficient to infer causation for such a complex disorder.

It is unlikely that one or even a few specific environmental agents are responsible for the majority of ASDs. It is more likely that some individuals have enhanced susceptibility to insults from the environment that may, in combination with their genetic predisposition, lead to autism. It is rarely possible to distinguish these complex relationships by simply evaluating trends in the general population.

The much publicized concern over vaccines and autism has primarily been based on such ecologic trends. More rigorous studies evaluating vaccine-related hypotheses are needed to incorporate individual-level exposure data, account for alternate exposures to metals, and evaluate susceptible subgroups of the population. However, attention should also be given to other environmental hypotheses.

Other environmental exposures found to be associated with autism include thalidomide, valproic acid, and infections such as rubella (Arndt et al. 2005; Chess 1971). These relatively rare exposures have been evaluated in small studies that have reported subtle effects. Yet, such findings support the plausibility that exposure to an environmental agent during a critical window of development can be associated with development of an ASD. The characteristic traits of autism are rarely distinguished before 2–3 years of age, but the cascade of events that leads to autism probably occurs much earlier, most likely during early gestation. Research focused on environmental exposures during critical periods of neurodevelopment should be prioritized.

Little is currently known about the etiology of autism, except that it is complex and multifactorial. The interaction between genetic and nongenetic factors during critical periods of neurodevelopment warrants further investigation. Until specific susceptibility genes are discovered, the identification of environmental risk factors that primarily affect susceptible subgroups may require us to refine ASD subgroup classifications using specific phenotypic patterns or the clustering of ASDs in families.

Given the complexity of autism, we will not find a magic bullet (genetic or environmental) to blame for most cases. There are probably many combinations of genes and environmental factors that contribute to the constellation of autistic traits. Future investigations of hypotheses involving environmental exposures need to carefully characterize cases, improve exposure assessment, focus on critical windows of neurodevelopment, and ensure sufficient power to conduct subgroup analyses and assess interactions. These considerations have been accommodated in a few well-planned epidemiologic studies that are, or soon will be, in progress. As we await advances in genetic and behavioral research, these studies offer hope for advancing our understanding of the potential role environmental factors play in the development of autism.

## Figures and Tables

**Figure:**